# Robust analyses for radiographic progression in rheumatoid arthritis

**DOI:** 10.1136/rmdopen-2022-002543

**Published:** 2023-04-04

**Authors:** Robert Landewé, Luna Sun, Yun-Fei Chen, Mo Daojun, Desirée van der Heijde

**Affiliations:** 1 Clinical Immunology & Rheumatology, University of Amsterdam, Amsterdam, Netherlands; 2 Rheumatology, Atrium Medical Centre, Heerlen, Netherlands; 3 Biomedicines, Eli Lilly and Company, Indianapolis, Indiana, USA; 4 Rheumatology, LUMC, Leiden, Netherlands

**Keywords:** Rheumatoid Arthritis, Cytokines, Arthritis, Juvenile

## Abstract

Demonstrating inhibition of the structural damage to joints as a statistically significant difference in radiographic progression as measured by the van der Heijde modified Total Sharp Score (mTSS) is a common objective in trials for rheumatoid arthritis treatments. The frequently used analysis of the covariance model with missing data imputed using linear extrapolation (analyses of covariance, ANCOVA+LE) may not be ideal for long-term extension studies or for paediatric studies. The random coefficient (RC) model may represent a better alternative.

A two-arm (active treatment and placebo) setting with a week 44 study period was considered. RC model, ANCOVA+LE and ANCOVA with last observation carried forward imputation were compared under different scenarios in bias, root mean square error (RMSE), power and type I error rate.

The RC model outperformed ANCOVA+LE in metrics measuring bias, RMSE, power and type I error rate under the evaluated scenarios. ANCOVA and RC provide similar performance when there are no missing data. With missing data, RC+observed (OBS) provides similar or better results than ANCOVA+LE in power and bias.

Our simulations support that RC is both a more sensitive and a more precise alternative to the commonly used ANCOVA+LE as a primary method for analysing mTSS in long-term extension and paediatric studies with a higher likelihood of missing data. The RC model can provide a reference at time points with missing data by estimating a slope; mTSS change by one unit change in time. ANCOVA+LE is recommended as a sensitivity analysis.

**WHAT IS ALREADY KNOWN ON THIS TOPIC**

- An important goal of randomised clinical trials on rheumatoid arthritis treatments is achieving a statistically significant reduction in radiographic progression of joint destruction between the active treatment group and the placebo control group. The frequently applied analyses of covariance models (ANCOVA) with linear extrapolation or last observation carried forward might not be sufficient to minimise bias and achieve more desirable precision when data become missing after patients took X-rays between the scheduled visit time points (out of the scheduled visit time window), switched to a rescue treatment or discontinued from study earlier. The missing data, especially data collected out of the scheduled visit time window, are more challenging as they become more frequent in the long-term extension and paediatric clinical trial settings. To overcome the analysis and interpretation challenges of missing data, we want to know whether a random coefficient (RC) model can perform better than ANCOVA through simulations and if the RC model would allow flexibility regarding when subject data must be collected.

**WHAT THIS STUDY ADDS**

- In this study, we report that RC and ANCOVA+linear extrapolation have equivalent performance for statistical power and bias when there is no missing data. The RC model has greater performance with missing data, relative to ANCOVA models.

**HOW THIS STUDY MIGHT AFFECT RESEARCH, PRACTICE OR POLICY**

- This study will provide recommendations for analytical methods in studies where there is a large gap between the scheduled visit and actual time of data collection, or where data are collected at different times for individual subjects, such as long-term extension and paediatric studies in which relatively high levels of missing data are likely to occur. The RC model provides a better opportunity to reduce bias and improve precision in evaluation of reduction in radiographic progression of joint destruction by an investigational study drug compared with the ANCOVA model.

## Introduction

Rheumatoid arthritis (RA) is a systemic, autoimmune, inflammatory disease, characterised by inflammation of the joints that causes pain, swelling, stiffness as well as progressive damage to the afflicted joints. Recently, management of RA has made significant improvements in treatment outcomes. The recommended primary target for the treatment of RA is a state of clinical remission.^1 2^ Although most patients still fail to achieve a state of sustained remission (remission lasting greater than 6 months).^3^ Complete and sustained remission is required to arrest further degradation of the joints: persistent joint inflammation leads to progressive joint destruction manifested by cartilage loss, erosive damage to juxta-articular bone and resultant functional impairment.^4 5^

Minimising structural damage is an important treatment goal for patients with RA. Achieving a statistically significant reduction in radiographic progression compared with standard of care (assessed by van der Heijde modified Total Sharp Score (mTSS)) is a common objective in clinical trials for new RA treatments. Multiple efforts (such as reminders sent to the patients for timely visits according to schedule, quality control in X-ray photo taking and reading, and clinical data entry, review, and cleaning) are planned and implemented in clinical trials to maximise avoidance of missing values. Yet, complete collection of radiographic data is an unrealistic goal, especially in longitudinal studies where patients who might initially respond well to a specific RA treatment become refractory over time to that treatment, and subsequently switch to a new treatment or discontinue from the study due to the loss of efficacy. Even with the most effective treatments available, such as TNF inhibitors, more than half of patients do not achieve a substantial response (primary failures) defined as either a 50% response rate in the American College of Rheumatology criteria ACR50) or achievement of low disease activity,^6 7^ and adherence to biological treatment is only approximately 60% over a period of 1–2 years, thus frequently necessitating a therapy switch.^8^

Even with data collected, the collection time may be off-visit-window in long-term extension studies, or just collected at a convenient time to limit exposure to X-ray as often seen in paediatric studies.

A frequently used analysis method for mTSS is the analysis of the covariance model with missing data imputed using linear extrapolation (analyses of covariance, ANCOVA+LE). In this method, the analyses of individual time points are carried out independently after imputation, that is, analyses are done by individual visits. However, this method might not be sufficient to minimise bias and achieve more desirable precision when there is a large gap between the scheduled visit and the actual time of data collection, or when data are collected at different times for individual subjects.

The random coefficient (RC) model is a special case of the linear mixed effect model, sometimes referred to as the linear mixed regression model. In this model, besides other covariates as in ANCOVA, time is also considered as a covariate.^9^ The coefficient for time (slope) is considered as a random sample from some population of possible coefficients, where the population of coefficient is defined by RA treatment. It differs from the ANCOVA model as it considers multiple time points simultaneously and does not require each subject to have their data collected at the same visits. It can also incorporate the exact time of data collection into the model. Both give flexibility for data collection, which may encourage enrolment or retention.

In this study, we have used a simulation to evaluate the RC model vs the ANCOVA+LE model on analysing structural damage measured by mTSS in patients with RA. Here, we have described the simulated trial, the measure of radiographic joint damage progression (ie, mTSS change from baseline), the simulated datasets for comparison between the RC model and ANCOVA model, and we present results from the modelling comparison. As well, we have described a case study from real clinical trial data analyses.

## Methods

### Simulation of a study that compares radiographic joint damage progression between treatment groups

Our simulated trial data mimics completed RA clinical trials, RA-BEAM and RA-BEGIN (NCT01710358 and NCT01711359),^10 11^ and a juvenile idiopathic arthritis trial (NCT03773978) on baricitinib (unpublished). The objective of the simulated study was to compare the progression of radiographic joint damage between the active treatment groups and the placebo treatment groups at weeks 12, 28 and 44 as this was when X-ray evaluation was scheduled in the juvenile idiopathic arthritis trial. The measure used for radiographic joint damage was the van der Heijde mTSS that quantifies the extent of bone erosion and joint space narrowing for 44 and 42 joints within the hands and feet, respectively, with higher scores representing greater damage.^12^ The radiographic joint damage progression for individual patients is measured by the mTSS change from baseline. The treatment effect (mTSS difference between treatment arms) is determined according to real RA clinical trials where the active treatment arm demonstrated a statistically significant difference compared with the placebo arm.^10 11^ We evaluate if RC is better than ANCOVA in terms of bias, root mean square error (RMSE), power and type I error rate under the simulated scenarios that consider the combinations of the relevant factors (mTSS baseline distribution, mTSS change over time, proportion of patients with progression, missing mTSS data at scheduled visits and sample size).

### Preparation of ‘full’, ‘observed’, ‘last observation carried forward imputation’ and ‘LE’ datasets for comparison between RC model and ANCOVA model

A ‘full’ dataset is a complete one without missing data. This dataset sets a benchmark scenario both for the commonly used ANCOVA, and for our proposed RC, to generate unbiased statistical inference with precision. However, this dataset cannot represent real clinical trials, as a ‘full’ dataset is almost impossible to achieve in a randomised clinical trial setting. The subject-level mTSS data at baseline are simulated to follow a log-normal distribution with a mean 1.55 and SD 1.35 that are consistent with the completed trials on baricitinib. The mTSS changes at postbaseline visits (week 12, 28 and 44) are simulated under linear, concave quadratic (fast progression then slow progression), and convex quadratic (slow progression then fast progression) assumptions (see an illustration in online supplemental SI figure 1). A wide range of possible progression (30%, 32% and 40% of the patients) reflects the possible long-term trial experience. The simulated difference in mTSS changes at week 44 between treatment groups is set from 0 to 0.9 to study a wide range of possible treatment differences. A sample size ranging from 150 to 700 per arm is simulated at the visits scheduled for X-ray evaluation.

10.1136/rmdopen-2022-002543.supp1 Supplementary data

Methodology on the statistics details of simulation for the subject-level mTSS dataset (ie,;full’ dataset) is provided in online supplemental file 1.

The ‘observed’ dataset (a ‘full’ dataset that removes missing data) mimics real clinical trials and is used to show if our proposed RC model is better than ANCOVA. The missing mTSS data follows a plausible monotone pattern (if one visit is missing, the sequential visits will be also missing). The monotone missing data are appropriate to simulate when patients are switched from an assigned treatment group (active treatment or placebo) to a rescue treatment, or when patients discontinue from a study. The missing data are simulated to follow a multinomial distribution where the accumulative missing rates vary by time points (5% at week 12, 15%%–35% at week 28 and 45%–60% at week 44). The wide range of missing data is simulated to evaluate the impact of overall missing rate and early missingness. Methodology on statistics details of missing data simulation is provided in online supplemental appendix.

‘LE’ and last observation carried forward imputation (‘LOCF’) datasets are generated by imputing the missing data from the ‘observed’ dataset, with LE method and last observation carried forward (LOCF) method. These two datasets are simulated for conducting analyses with the ANCOVA model that is commonly used for radiographic joint damage progression evaluation. Methodology on the statistical details of simulating the ‘LE’ and the ‘LOCF datasets is provided in online supplemental appendix.

### Analysis of simulated datasets for comparison between RC and ANCOVA

To tackle the real-world missing data issue in radiographic joint damage progression, we propose using the RC model to analyse the ‘observed’ dataset. In the RC model, baseline mTSS, treatment, time and time-by-treatment interactions are fixed effects, and time is a random effect. Given that the key interest is in the longitudinal profile for individual patients and treatment, we used a random slope model with parameter for time as the slope, and this allows us to analyse multiple time points simultaneously in the RC model. Of note, given that the random slope model is a form of linear mixed effect model, inference is valid only under the missing at random mechanism which is plausible in evaluation of radiographic structural changes in RA. We also analysed the ‘full’ dataset with the RC model to serve as a benchmark of valid statistical inference with precision. For comparison, we analysed ‘full’, ‘LE’ and ‘LOCF’ datasets with the ANCOVA model when baseline and treatment are independent variables. Details of the statistical analyses are presented in table 1.

**Table 1:** Analysis methods

Method abbreviation	Analysis model	Imputation method for missing data	Comments
ANCOVA+LOCF	ANCOVA	LOCF	A common sensitivity analysis
RC+OBS	RC	None	New proposed analysis
ANCOVA+LE	ANCOVA	LE	Current standard analysis
RC+FULL	RC	Not applicable	Ideal scenario as benchmark, not representative of real trials. Results expected to be similar to ANCOVA+FULL
ANCOVA+FULL	ANCOVA	Not applicable	Ideal scenario as benchmark, not representative of real trials. Results expected to be similar to RC+FULL

ANCOVA+LOCF: ANCOVA model using LOCF imputed data.

RC+OBS: RC model using observed dataset with missing data.

ANCOVA+LE: ANCOVA model using LE imputed data.

RC+FULL: RC model using full dataset without missing data.

ANCOVA+FULL: ANCOVA model using full dataset without missing data.

A summary of the steps used in each simulation is shown in online supplemental file 2.

ANCOVA, analyses of covariance; LE, linear extrapolation; LOCF, last observation carried forward; RC, random coefficient.

10.1136/rmdopen-2022-002543.supp2 Supplementary data

From each of the analyses, the difference in change from baseline of mTSS between active treatment and placebo treatment groups is estimated for statistical inference. This is repeated 500 times under each simulation scenario with different combinations of the relevant parameters (ie, mTSS baseline distribution, mTSS change over time, proportion of patients with progression, missing mTSS data at postbaseline scheduled visits, and sample size). The details of these parameters are given in online supplemental appendix. Briefly, we compared bias, RMSE, power and type I error rate between the RC and ANCOVA models under each simulation scenario. Details of the statistical analysis are given in online supplemental appendix.

## Results

**Figure 1:**
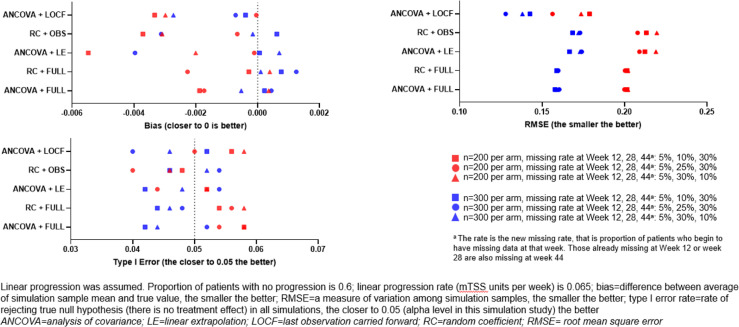
Assessment of analysis methods with simulated progression data (no treatment difference and linear progression).

The effect of missing data on the robustness of analysis methods was assessed following simulation of the data and analysis using the RC and ANCOVA models, using both the observed (OBS) and imputed datasets (LE and LOCF). The effect of the different methods used on data with no treatment difference and linear progression is shown in figure 1. In this situation, all analysis methods delivered unbiased, robust results, with type 1 error rates close to the nominal levels. As expected, differences in RMSE were observed following changes in sample size, with a larger sample size resulting in a lower RMSE. Similar trends were observed under quadratic progression assumptions (data not shown).

**Figure 2:**
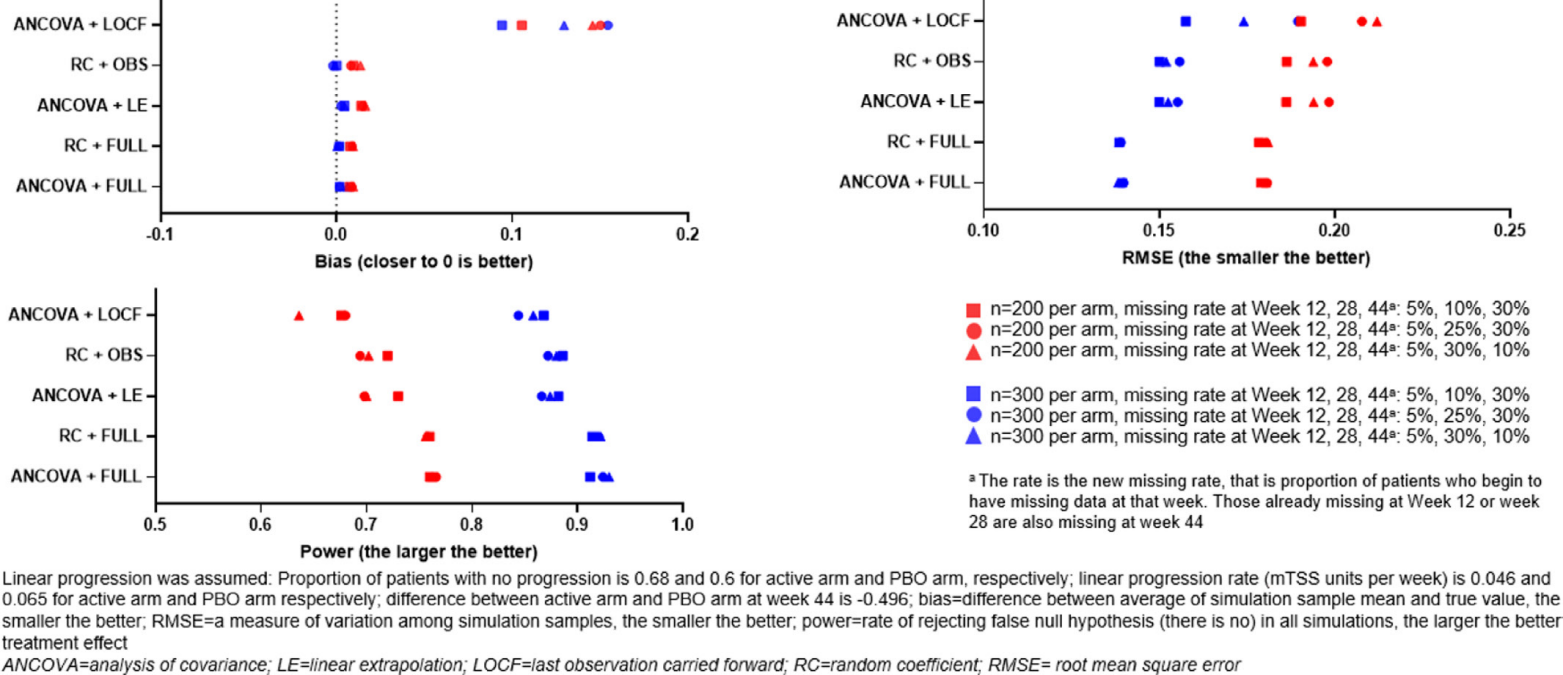
Assessment of analysis methods with simulated progression data (slowed progression in active arm and assumed linear progression).

**Figure 3:**
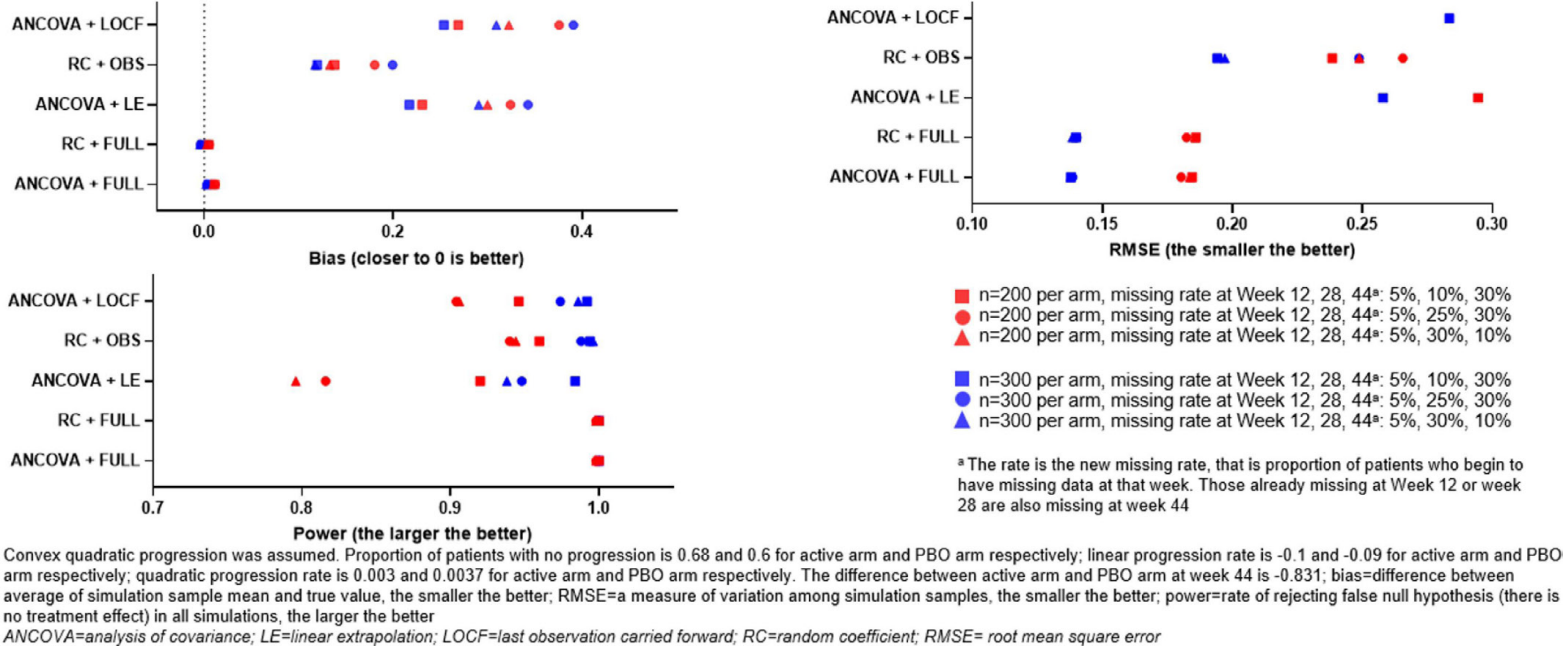
Assessment of analysis methods with simulated progression data (slowed progression in active arm and assumed convex quadratic progression).

Figure 2 shows the effect of different analysis methods in the simulated scenario of linear progression, with the ‘active’ arm causing a slowing of progression (ie, the intervention is displaying efficacy). In this scenario, the ANCOVA+LOCF method displayed a notably higher level of bias. Both RC+OBS and ANCOVA+LE methods displayed a similar level of RSME, bias and statistical power. The trend of higher sample sizes resulting in smaller RMSE values and higher statistical power was observed in all analysis methods assessed. The effect of non-linear progression on the different analysis methods was more pronounced. As shown in figure 3, assessment of the analysis methods with assumed convex quadratic progression led to a large degree of observed bias where data was missing, and a pronounced effect on RMSE and statistical power: in cases of missing data, RC+OBS displayed the smallest bias, followed by ANCOVA+LE. RC+OBS also displayed a smaller RMSE and larger statistical power compared with the other analysis methods used. RC+FULL and ANCOVA+FULL were unbiased, and had smaller RMSE and greater statistical power.

**Figure 4:**
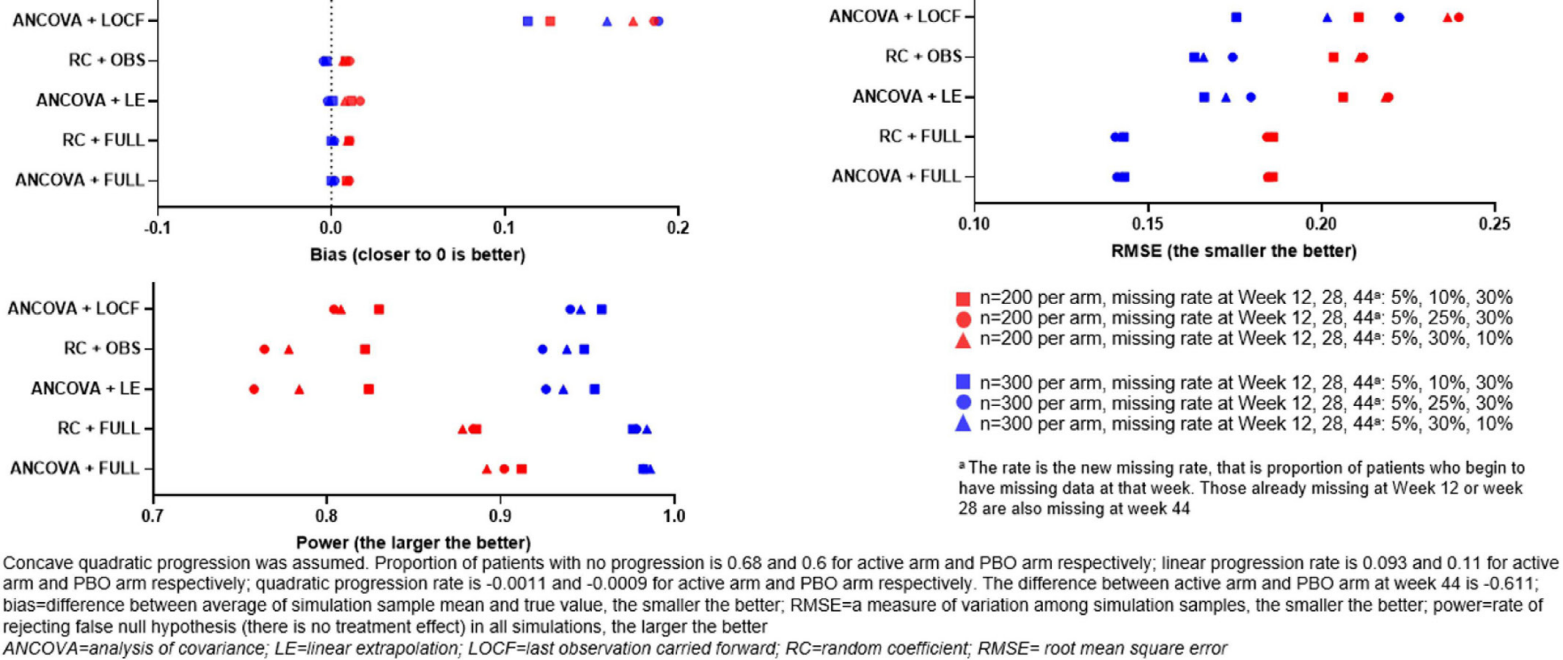
Assessment of analysis methods with simulated progression data (slowed progression in active arm and assumed concave quadratic progression).

Assessment of the analysis methods with assumed concave quadratic progression (figure 4) demonstrated similar effects as concave progression for the ANCOVA+LOCF method; however, the RC+OBS and ANCOVA+LE methods displayed similar performance overall.

Baricitinib is an oral selective inhibitor of Janus kinase (JAK) 1 and JAK 2 that has been approved for the treatment of moderately to severely active RA in adults. RA-BEAM (NCT01710358) was a randomised, double-blind, double-dummy, placebo-controlled and active-controlled, parallel-arm, 52-week study conducted at 281 centres in 26 countries. Patients enrolled in RA-BEAM had an inadequate response to methotrexate and were randomised 3:3:2 to placebo once daily, baricitinib 4 mg once daily or adalimumab 40 mg biweekly.^11^ X-ray data were scheduled at screening, week 16, week 24 and week 52, with week 24 as primary evaluation time point. X-ray data were only taken at early termination if the most recent X-ray was more than 12 weeks earlier. To reduce imputation and use available data as much as possible, postbaseline X-ray administered within 14 days prior to the scheduled visit data, or within 30 days after the scheduled visit date were used as if scheduled. The LE method was prespecified as the primary imputation method for X-ray data to impute missing data at time points when analyses were conducted (including the analyses at week 24 and week 52). LOCF was also prespecified as an alternative imputation method for missing data.

We used X-ray data from RA-BEAM as a case study to illustrate the use of ANCOVA+LE, ANCOVA+LOCF and RC+OBS methods. For the RC+OBS method, we also considered three ways of incorporating time information: (1) use scheduled week (week 24) and baseline week=week 0; (2) use scheduled week (week 24) and baseline week=−3.3 (average of actual relative week to randomiSation); (3) use day (actual relative day to day of randomisation) and baseline day=0. Results are summarised in table 2.

**Table 2:** Case study from RA-BEAM on mTSS analyses

Model	Base time	Label	Treatment difference at week 24	P value	Slope estimate
ANCOVA+LE	Week=0	BARI-4mg vs PBO	−0.4890	<0.0001	.
ANCOVA+LOCF	Week=0	BARI-4mg vs PBO	−0.4514	<0.0001	.
RC+obs	Week=0	BARI-4mg vs PBO	−0.6206	<0.0001	−0.026
RC+obs	Week=−3.3	BARI-4mg vs PBO	−0.5141	<0.0001	−0.021
RC+obs	Day=0	BARI-4mg vs PBO	−0.4894	<0.0001	−0.020

ANCOVA, analysis of covariance; BARI, baricitinib; LE, linear extrapolation; LOCF, last observation carried forward; obs, observed; PBO, placebo; RA, rheumatoid arthritis; RC, random coefficient.

In all methods, a statistically significant decrease in the progression of mTSS was observed for the baricitinib group for the study duration. RC+OBS, with day relative to randomisation as the time variable, provided a similar estimate for ANCOVA+LE. It also provided slope estimate that could be used for time points without data if we continue to assume linear progression.

## Discussion

Here, we used systemic simulation to examine the performance of the RC model. The following observations were made, in cases with no treatment effect, all methods were generally unbiased, with similar type 1 error rates close to the nominal level. ANCOVA and RC provide similar performance when there is no missing data. However, when data are missing, RC+OBS provided better or at least equivalent performance as ANCOVA+LE in power and bias. As expected, the more missing data in early visits, the larger bias, the smaller power and the larger RMSE. ANCOVA+LOCF have much larger bias in linear (figure 2), and concave quadratic (figure 3) change patterns compared with ANCOVA+LE and RC+OBS. When change patterns are convex quadratic (figure 4), all models have large bias when data are missing. ANCOVA+LOCF had the largest bias whereas RC+OBS had the smallest bias, followed by ANCOVA+LE. Our simulations support the hypothesis that the RC model is superior to LOCF. In fact, it is known from the literature that LOCF can yield bias, and it may even yield bias if data are missing completely at random.^13 14^

There are several limitations in this work. First, our simulations were conducted under a specific assumption of monotone missing pattern. Therefore, the RC model may not be sufficiently robust to fit all possible scenarios. However, this assumption fits well in the most common missing data scenario in RA trials where patients would be considered to have missing data after switching to the rescue treatment for clinical improvement, or after dropping out of the trial. In RA-BEAM (the case study), among those patients who had missing data, 84% were monotonic. Although we have not studied nonmonotone missingness patterns, it is known from the literature that linear mixed models generally give unbiased results for monotone as well as nonmonotone missingness patterns, as long as the data are missing at random.^15^ Furthermore, RC model itself is sufficiently robust to handle the data that are missed at the target time point but are collected at alternative time points. For example, if a patient missed a scheduled X-ray visit but took a make-up X-ray shortly after the missed visit, before the next scheduled X-ray visit. Second, the current model only assumes linear progression in modelling. However, we generated the data from a quadratic progression assumption to investigate the robustness of the RC model, and this model appeared to be robust. While a more flexible model may be implemented to have a random quadratic coefficient, such complexity may not be necessary given the small bias and reasonable power observed with a linear coefficient. An even more saturated linear mixed effect model such as mixed model repeated measure (MMRM), in which time is considered a factor, may also be used to model X-ray data. This model can estimate all types of change assumption, such as worsening then improved (less damage compared with before) or improved then worsening (more damage compared with before). However, MMRM does not have the flexibility of the RC model because it requires the data collection visit window to be the same for individual subjects, and does not allow inference in time points when no data were collected. Conversely in the RC model, the estimated coefficients may be used to estimate treatment effect at time points when no data were collected. Furthermore, the nature of RA disease progression may not allow ‘improved’ structure over time. While negative values could be observed due to measurement error, a more complicated structure may not be necessary. Third, we assume the active treatment and placebo arm have the same missing data rate. A more complex scenario can be considered with two arms having different missing data rates and different missing mechanism. Last, our simulations were conducted with commonly used statistical methods (ie, we make comparisons between the RC model and other commonly used models). Applying less commonly used methods may further enhance statistics precision. Some studies have demonstrated that constrained analysis increases precision as compared with ANCOVA under the assumptions of missing at random and no systemic difference in baseline measures of interest.^16 17^ Of note, our longitudinal evaluation of radiographic structure in RA is likely to meet these assumptions because it is not likely to have systematic differences at baseline between treatment groups in blinded randomised controlled clinical trials, and the most hypothesised missing at random is plausible in radiographic progression evaluation. Given that the random slope model is a form of linear mixed effect model, inference is valid only under the missing at random mechanism which is plausible in evaluation of radiographic structural changes in RA.

## Conclusion

The results presented here support that RC+OBS is both more a sensitive and more a precise alternative to the commonly used ANCOVA+LE as a primary method for analysing mTSS in long-term extension and paediatric studies with a higher likelihood of missing data. The RC model can also provide a reference for time points when no data are collected via the estimated slope. The current standard method, the ANCOVA+LE model, can be used for sensitivity analysis, however, ANCOVA+LOCF is not recommended as it has higher bias and lower power than other methods for the assessments.

## Data Availability

Data is available upon reasonable request. Eli Lilly and Company provides access to all individual participant data collected during the trial, after anonymisation, with the exception of pharmacokinetic or genetic data. Data are available to request 6 months after the indication studied has been approved in the United States and the European Union and after primary publication acceptance, whichever is later. No expiration date of data requests is currently set once data are made available. Access is provided after a proposal has been approved by an independent review committee identified for this purpose and after receipt of a signed data sharing agreement. Data and documents, including the study protocol, statistical analysis plan, clinical study report and blank or annotated case report forms, will be provided in a secure data sharing environment. For details on submitting a request, see the instructions provided at www.vivli.org.
