# Supplementary material for: Robust analyses for radiographic progression in rheumatoid arthritis
Source: RMD Open. 2023 Apr 4;9(2):e002543. doi: 10.1136/rmdopen-2022-002543 (PMC10083852; doi:10.1136/rmdopen-2022-002543)
Supplement: Supplementary data [file rmdopen-2022-002543supp001.pdf]

BMJ Publishing Group Limited (BMJ) disclaims all liability and responsibility arising from any reliance placed on this supplemental material which has been supplied by the author(s)

Supplementary information

Table of contents

**Page 2**	**Simulation Setup and Scheme**
**Page 4**	**SI Table 1: Metrics of Comparison**
**Page 5**	**SI Table 2: Key simulation parameters**
**Page 6**	**SI Figure 1. Illustration of progression patterns used in the model**
**Page 7**	**SI Figure 2: Examples of individual simulation scenarios**

Landewé R, *et al. RMD Open* 2023; 9:e002543. doi: 10.1136/rmdopen-2022-002543

Simulation Setup and Scheme

Simulate subject-level data

The simulation was used to compare analysis models and imputation approaches. The complete simulation flow is illustrated in Figure 1 and explained in detail below. In general, we consider a 2-arm (active treatment and placebo) clinical trial setting. A log-normal distribution was used to generate baseline subject-level mTSS data. Changes in mTSS (at week 12, 28, and 44) were generated under linear, concave quadratic (fast progression then slow progression), and convex quadratic (slow progression then fast progression) assumptions (see an illustration in SI Figure 1), with the proportion of change forced to be 0 (a proportion of simulated subjects do not have progression). A monotone missing pattern was assumed to generate a data set with missing data. Parameters used for generating simulated data were obtained based on observed baseline and change from baseline in mTSS from recently completed clinical trials (RA-BEAM and RA-BEGIN, NCT01710358 and NCT01711359)[11, 14]. The parameter value for the simulation scenario are summarized in Table 1.

Simulation details

1. Active treatment and placebo arm subjects share common baseline distribution as, in clinical trials, subjects would be randomized, and there is no difference expected between arms. Random numbers from $lognormal(\mu, \sigma^2), \mu$ and σ correspond to row one in Table 2, and were generated to present the baseline mTSS score.
2. Let $i = 1$ present active treatment arm, $i = 2$ present placebo arm, $j = 1\ to\ n_i$ present j^{th} subject from treatment i. Notice that n_1 can be the same or different from n_2. Also, let $t = 12, 28, 44$ present visit week.

   a) Progression assumption: For linear progression, change scores at time t were generated as random numbers follow zero-inflated normal distribution: with probability p_i, change scores were forced to 0 (corresponding to the proportion of patients without progression); with a probability $1 - p_i$, change scores were generated from a normal distribution with mean $\beta_i \times t$ and standard derivation $t^2 \times (\sigma_a^2) + \sigma_e^2$. β_i is fixed effect of average progression rate for group i. a_j is random effect for subject specific progression rate following normal distribution of mean 0 and standard deviation σ_a. ϵ_{ijt} is time specific random error, which also follows a normal distribution with mean 0 and standard deviation σ_e. a_j and ϵ_{ijt} are independent.

$$Chg(T = t) = \begin{cases} 0 & \text{with prob } p_i \\ (\beta_i + a_j) * t + \epsilon_{ijt}, a_j \sim N(0, \sigma_a^2), \epsilon_{ijt} \sim N(0, \sigma_e^2),\ a_j\ and\ \epsilon_{ijt}\ are\ independent & \text{with prob } 1 - p_i \end{cases}$$

   b) Quadratic progression assumption: For quadratic progression, change scores at time t were generated as random numbers follow zero-inflated normal distribution: with probability p_i, change scores were forced to 0; with a probability $1 - p_i$ change scores were generated from a normal distribution with mean $\alpha_i \times t^2 + \beta_i \times t$ and standard deviation $t^2 \times \sigma_a^2 + \sigma_e^2$. α_i is fixed effect representing average quadratic term for progression rate for group i, β_i fixed linear term for average progression rate for group i. a_j is random effect for subject specific progression rate following normal distribution of mean 0 and standard deviation σ_a. ϵ_{ijt} is time specific random error,

which also follows a normal distribution with mean 0 and standard deviation σ_e. a_j and ϵ_{ijt} are independent. With different values of α, the overall progression can be concave ($\alpha < 0$) or convex ($\alpha > 0$).

$$Chg\ (T=t) = \begin{cases} 0 \text{ with prob } pi \\ (\alpha_i) * t^2 + (\beta_i + a_j) * t + \epsilon_{ijt}, a_j \sim N(0, \sigma_e^2),\ a_j\ and\ \in_{ijtt}\ are\ independent \text{ with pro } 1 - p_i \end{cases}$$

With these model assumptions, each subject has the same slope for each time point; however, each timepoint has its own error term.

To make the generated data mimic real possible mTSS scores (evaluated by two evaluators independently each with 448 as possible maximums, then averaged), we rounded the simulated mTSS score at baseline and at timepoint t (= simulated baseline + simulated change at time t) to the closest 0.5, and truncated the value between (0, 448). We called these values the adjusted baseline score and adjusted time t score. A new change score at time t with 0.5 as potential increment was recalculated as the difference between adjusted time t score minus adjusted baseline score. This dataset is called "Full" dataset, which only exists in ideal scenarios. ANCOVA and RC model were applied on this full dataset to serve as benchmark for simulation comparison.

Simulation of missing data scenario

This step created an 'observed' data set to reflect missing data scenario. We assumed monotone missing pattern (i.e. if one visit was missing, the sequential visits were also missed) and used multinomial distribution to generate missingness. The probabilities in the distribution are not equal and set differently based on the simulation.

Assuming no missingness at baseline, we generated a random number from $multinomial(1,\ b_1, b_2, \ldots, b_K)$ with $\sum b_i = 1$, $K =$ max of visit, here is 4 (baseline, week 12, week 28, and week 44). b_1 is the probability of missingness starting from visit 2, b_2 is the probability of missingness starting from visit 3, b_{K-1} is the probability of missingness starting from last visit (visit K). b_K is the probability of having complete data. If that random number is 1, then the subject would have no post-baseline data; if that number is 2, then the subject would only have baseline and week 12 data; if that number is 3, the subject would have baseline, week 12, and time week 28 data; if that number if 4, the subject does not have missing data. We repeated this for each individual subject.

Impute "observed" dataset to create "imputed" dataset

Missing data in "Observed" dataset were imputed with linear extrapolation imputation or last observation carried forward imputation (LOCF) methods to create 'LE' dataset and 'LOCF' datasets, respectively.

a. LE dataset: at week x, impute missing mTSS score as $LE_{mTSS} = baseline + (x - 0) \times (AVAL_{recent} - AVAL_{baseline}) \div (WK_{recent} - 0)$ where WK_{recent} and $AVAL_{recent}$ are the closest week with available mTSS data collected to week x and the corresponding mTSS value.
b. LOCF dataset: at week x, impute missing mTSS score as $LOCF_{mTSS} = AVAL_{recent}$ where $AVAL\_recent$ is the available data that were obtained at the time closest to week x.

Step 2.1.1 to 2.1.4 would generate one simulation data cohort ("Full" dataset, "observed" dataset, "LE" dataset, and "LOCF" dataset). SI Figure 2 gives an illustration of subject-level simulated data for this data cohort. A summary of the steps used in each simulation is shown in Figure 1 below.

Analyses of simulated subject-level data

A summary of the analysis methods used is presented in Table 2. The primary method of interest is the random coefficient (RC) model without imputation. In this model, baseline, treatment, time, and time-by-treatment interactions are fixed effect and time is a random effect. We also analyzed the "Full" dataset with the RC model to serve as a benchmark for other methods. Again, multiple time points are analyzed simultaneously in the RC model. Another model of interest is the ANCOVA model. It was applied on the "LE" dataset, the "LOCF" dataset, as well as the "Full" dataset using baseline and treatment as predictors.

From each analysis, the difference in change from baseline of mTSS between active treatment and placebo was estimated, and hypothesis testing on whether there was a difference between active treatment and placebo was performed. These steps were repeated 500 times under each simulation scenario (combinations of parameters in Table 2). We compared bias, root mean square error (RMSE), power and type I error rate among methods under each simulation scenario. More details about these metrics can be found in Table 1 and SI Table 2.

SI Table 1: Metrics of Comparison

**Metric of Comparison**	**Definition**	**Interpretation**
Bias	Difference between average of simulation sample mean and true value	The closer to 0, the better
RMSE	A measure of variation among simulation samples	The smaller, the better
Type I error rate	Number of wrong rejections of null hypothesis (two arms are the same) when they are the same	The closer to nominal value (alpha=0.05 in this work), the better
Power	Number of correct rejections of null hypothesis (two arms are the same) when they are different	The larger, the better

SI Table 2: Key simulation parameters

Parameters		True Value	Comments
Mean and standard deviation in log-normal distribution in baseline value generation		1.55 and 1.35	Baseline mean mTSS was approximately 11.7
Sample size per study arm		150, 200, 300, 500, 700	To explore a wide range of sample sizes and different sample size combinations
Proportion of patients with no progression		0.6, 0.68, 0.7	To mimic real trial observed scenarios and a wide range of possible combinations
Difference in change from baseline in mTSS at Week 44 between active treatment and placebo		0 to -0.9	To investigate a wide range of realistic treatment differences
Cumulative missing rate at Week 12, Week 28, Week 44		(5%, 15%, 45%);<br>(5%, 30%, 60%);<br>(5%, 35%, 45%)	Proportion of completers ranges from 40% to 55%<br>To investigate impact of overall missing rate and early missingness

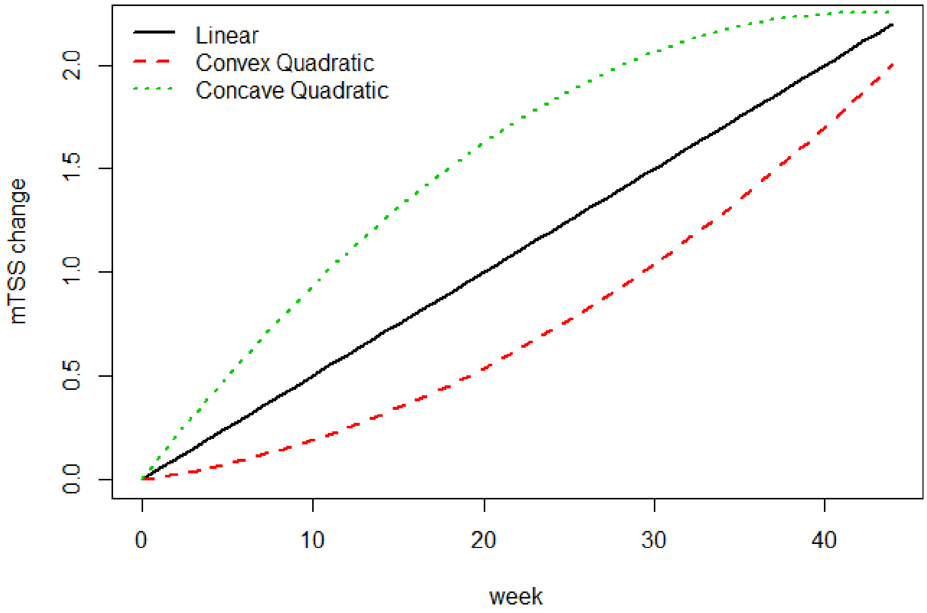

SI Figure 1. Illustration of progression patterns used in the model

Abbreviations: mTSS, modified total Sharp score

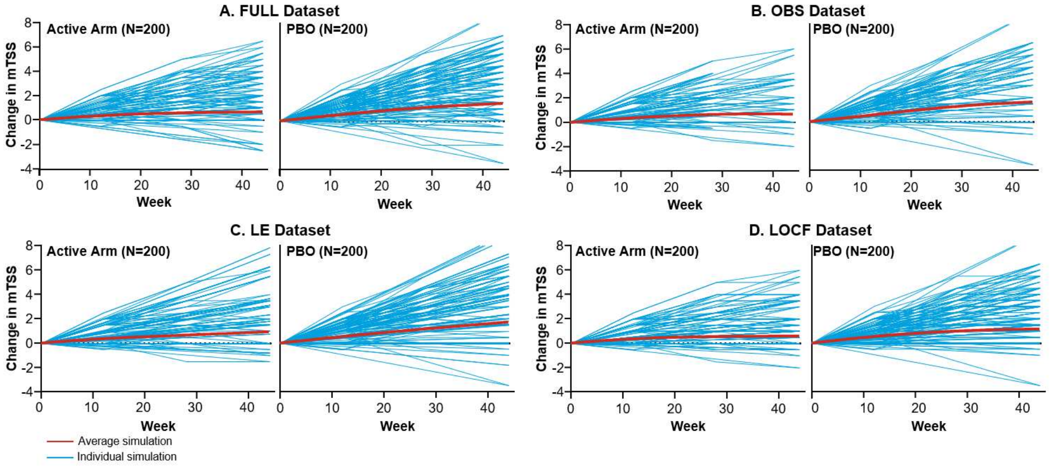

Concave quadratic progression was assumed in this illustration. Proportion of patients with no progression is 0.68 and 0.6 for active arm and PBO arm, respectively; linear progression rate is 0.093 and 0.11 for active arm and PBO arm, respectively; quadratic progression rate is -0.0011 and -0.0009 for active arm and PBO arm, respectively; quadratic progression rate is -0.001 and -0.0009 for active arm and PBO respectively. LE=Linear extrapolation; LOCF=last observation carried forward; Loess=locally estimated scatterplot smoothing; mTSS=modified total Sharp score; PBO=placebo. Blue lines represent individual simulations. Red lines represent the average of the simulations.

SI Figure 2: Examples of individual simulation scenarios
