# Supplementary material for: Robust analyses for radiographic progression in rheumatoid arthritis
Source: RMD Open. 2023 Apr 4;9(2):e002543. doi: 10.1136/rmdopen-2022-002543 (PMC10083852; doi:10.1136/rmdopen-2022-002543)
Supplement: Supplementary data [file rmdopen-2022-002543supp002.pdf]

‘Full’ Dataset

Simulate patient-level mTSS data over time, based on:

- Linear progression
- Quadratic progression
- A ‘forced’ group of patients with no progression overtime

Users parameter values were estimated from recently completed large Phase 3 RA clinical trials[5-7]

‘Observed’ Dataset and ‘Imputed’ Dataset

- Consider multiple missing data scenarios: Create patient-level data with missingness – ‘Observed’ dataset
- Impute “observed” data with 1) LE and 2) LOCF methods to create ‘LE’ dataset and ‘LOCF’ dataset (Figure 3)

Analyses Simulated Patient-level Data

- Apply ANCOVA on Full dataset, LE dataset, and LOCF dataset with baseline and treatment arm as predictors (Table 2)
- Apply RC model on Full dataset and Observed dataset (Table 2)

Compare Methods

- Compare methods on estimating difference between two treatment arms
- Look at bias, RMSE, type 1 error rate/power (Table 3) with 500 simulations

ANCOVA=analysis of covariance; LE=linear extrapolation; LOCF=last observation carried forward; mTSS=modified total Sharp score; RA=rheumatoid arthritis; RA=random coefficient; RMSE=root mean square error
